# Impact of Sodium Thiosulfate on Prevention of Nephrotoxicities in HIPEC: An Ancillary Evaluation of Cisplatin-Induced Toxicities in Ovarian Cancer

**DOI:** 10.1245/s10434-023-14216-6

**Published:** 2023-09-14

**Authors:** Rosemary N. Senguttuvan, Nicole Lugo Santiago, Ernest S. Han, Byrne Lee, Stephen Lee, Wei-Chien Lin, Mehdi Kebria, Amy Hakim, Jeff F. Lin, Mark T. Wakabayashi, Nora Ruel, Raechelle Tinsley, Melissa Eng, Daphne B. Stewart, Edward W. Wang, Benjamin I. Paz, Xiwei Wu, Hyejin Cho, Winnie S. Liang, Lorna Rodriguez-Rodriguez, Mihaela C. Cristea, Mustafa Raoof, Thanh H. Dellinger

**Affiliations:** 1 Division of Gynecologic Oncology, Department of Surgery, City of Hope Comprehensive Cancer Center (COH), Duarte, CA USA; 2 Department of Surgery, Stanford, Stanford, CA USA; 3 Regeneron Pharmaceuticals, Inc., Tarrytown, NY USA; 4 Biostatistics Core, City of Hope BRI, Duarte, CA USA; 5 Clinical Trials Office, COH, Duarte, CA USA; 6 Department of Medical Oncology, COH, Duarte, CA USA; 7 Division of Surgical Oncology, Department of Surgery, COH, Duarte, CA USA; 8 Integrative Genomics Core, City of Hope Beckman Research Institute (BRI), Duarte, CA USA; 9 Translational Genomics Research Institute, Phoenix, AZ USA

## Abstract

**Purpose:**

Hyperthermic intraperitoneal chemotherapy (HIPEC) with cisplatin confers a survival benefit in epithelial ovarian cancer (EOC) but is associated with renal toxicity. Sodium thiosulfate (ST) is used for nephroprotection for HIPEC with cisplatin, but standard HIPEC practices vary.

**Methods:**

A prospective, nonrandomized, clinical trial evaluated safety outcomes of HIPEC with cisplatin 75 mg/m^2^ during cytoreductive surgery (CRS) in patients with EOC (*n* = 34) and endometrial cancer (*n* = 6). Twenty-one patients received no ST (nST), and 19 received ST. Adverse events (AEs) were reported according to CTCAE v.5.0. Serum creatinine (Cr) was collected preoperatively and postoperatively (Days 5–8). Progression-free survival (PFS) was followed. Normal peritoneum was biopsied before and after HIPEC for whole transcriptomic sequencing to identify RNAseq signatures correlating with AEs.

**Results:**

Forty patients had HIPEC at the time of interval or secondary CRS. Renal toxicities in the nST group were 33% any grade AE and 9% grade 3 AEs. The ST group demonstrated no renal AEs. Median postoperative Cr in the nST group was 1.1 mg/dL and 0.5 mg/dL in the ST group (*p* = 0.0001). Median change in Cr from preoperative to postoperative levels were + 53% (nST) compared with − 9.6% (ST) (*p* = 0.003). PFS did not differ between the ST and nST groups in primary or recurrent EOC patients. Renal AEs were associated with downregulation of metabolic pathways and upregulation of immune pathways.

**Conclusions:**

ST significantly reduces acute renal toxicity associated with HIPEC with cisplatin in ovarian cancer patients. As nephrotoxicity is high in HIPEC with cisplatin, nephroprotective agents should be considered.

**Supplementary Information:**

The online version contains supplementary material available at 10.1245/s10434-023-14216-6.

Hyperthermic intraperitoneal chemotherapy (HIPEC) is the delivery of heated chemotherapy (42 °C) into the intraperitoneal (IP) cavity immediately after optimal cytoreductive surgery (CRS). The OVHIPEC-1, randomized, phase III trial evaluated HIPEC with cisplatin 100 mg/m^2^ in patients with epithelial ovarian cancer (EOC) and demonstrated an 11.8-month overall survival benefit for stage III patients undergoing interval cytoreduction and HIPEC.^1–3^ Cisplatin is considered the drug of choice in HIPEC for EOC due to its thermal enhancement at temperatures from 42 to 44 °C. It is renally excreted and, consequently, may result in significant renal toxicity.^4^ Other accepted HIPEC regimens in EOC include paclitaxel, cisplatin, oxaliplatin, cisplatin with doxorubicin, as well as cisplatin with mitomycin-C.^5^

The incidence of acute kidney injury (AKI) with cisplatin during HIPEC has been reported at 15–31% for cisplatin doses 50–100 mg/m^2^.^6,7^ Management of cisplatin-associated nephrotoxicity includes perioperative hyperhydration, mannitol, amifostine, and sodium thiosulfate (ST) administration.^6,7^ ST is an agent used to treat calciphylaxis, kidney stones, and uremic vascular calcification.^8–10^ The mechanism of action of ST regarding nephroprotection is unclear, and some concerns exist that ST use may reduce the cytotoxic effect of chemotherapy due to its anti-alkylating properties.^11^ ST was used as a pre-HIPEC bolus and as postoperative maintenance in all patients undergoing HIPEC in the OVHIPEC-1 trial, with few renal toxicities reported.^1^ Nonetheless, the use of nephroprotectants in association with HIPEC with cisplatin is variable, and use of ST in the United States is limited.^7,12^

Use of ST versus no ST (nST) has not been prospectively evaluated in ovarian cancer patients undergoing HIPEC with cisplatin. Rather, interim analyses showing higher-than-expected, renal, adverse effects has led to protocol amendment with inclusion of ST prophylaxis in some key HIPEC trials in ovarian cancer.^13,14^ Additionally, no studies have investigated the molecular changes in peritoneal tissues exposed to cisplatin during HIPEC. Investigation of these changes may help to identify patients at higher risk of HIPEC-induced nephrotoxicity. We report clinical and molecular results of a retrospective, ancillary study of EOC and endometrial cancer (EC) patients who underwent HIPEC with cisplatin at 75 mg/m^2^ with and without ST administration.

## Methods

### Study Population

A single-institution, clinical trial was approved by the City of Hope Institutional Review Board (NCT01970722) to evaluate feasibility and safety of HIPEC with cisplatin in EOC and EC patients. All research was carried out in accordance with ethical principles as defined by the Declaration of Helsinki.^15^

Eligible patients had newly diagnosed or recurrent stage III/IV EOC, primary peritoneal, fallopian tube cancer, or EC. Patients were required to have an Eastern Cooperative Oncology Group (ECOG) performance status of 0 or 1 and preexisting normal renal function. Patients were allowed to have received neoadjuvant chemotherapy before planned HIPEC. Complete or optimal gross resection of disease defined by R classification was required to be anticipated, as judged by participating surgeons.^16^ Written, informed consent was obtained before patient inclusion, and baseline demographics were obtained at enrollment. Patients were followed prospectively for evaluation of response to treatment, disease status, progression-free survival (PFS), and length of follow-up. Enrollment proceeded until the target patient number was accrued without triggering safety stopping rules (grade ≥ 4 morbidity rate ≥ 40%; mortality rate ≥ 3.4%).^17^ All subjects were recruited from patients undergoing treatment at City of Hope National Comprehensive Cancer Center (Duarte, CA). Supplementary Data 1 shows complete eligibility and exclusion criteria.

### Trial Design

This prospective, feasibility, clinical trial investigated the safety and molecular changes associated with HIPEC with cisplatin in patients with gynecologic malignancies. Initial protocol treatment did not include ST administration. However, after an interim analysis of the first 21 patients demonstrated a high rate of renal toxicity, the protocol was amended. ST was then added to the protocol for the subsequent 19 patients recruited. This study examines the effects of the protocol change in the trial.

OVHIPEC-1 was referenced for the ST dosing protocol. For patients receiving ST, a bolus of 9 g/m^2^ in 200 cc of sterile water was administered intravenously immediately before HIPEC. HIPEC with cisplatin was delivered at 75 mg/m^2^ over 60 min at 41–43 °C immediately after optimal CRS by using closed or laparoscopic methods, determined by surgeon preference. The ThermoChemHT-2000 (ThermaSolutions, White Bear Lake, MN) hyperthermic pump system was used. After completion of all cytoreductive surgical procedures, two inflow tubes were placed in the pelvic cavity, and two outflow tubes were placed in the upper abdomen with IP thermometer probes attached. The abdomen was temporarily closed. Saline solution was used to confirm smooth circulation to and from the HIPEC pump. Once target temperature and adequate circulation were reached, chemotherapy was introduced in one dose and circulated for 60 min, while gently shaking the abdomen side-to-side to achieve even distribution. Following completion of HIPEC with cisplatin, a maintenance dose of ST of 12 g/m^2^ in 1000 cc of sterile water was administered continuously over 6 h to patients in the ST group. In all instances, urinary output was closely monitored. Additional, intravenous, fluid boluses were administered to avoid urine output < 1 mL/kg perioperatively in both groups. Adverse events (AEs) were collected and graded according to CTCAE v.5.0.^18^

Normal peritoneal samples were obtained at the start of surgery (pre-HIPEC) and immediately after HIPEC. Samples were primarily collected from the abdominal, pelvic, or diaphragmatic peritoneum. Other sample origins that included the omentum, appendix, and adipose tissue were considered. All normal samples were collected from locations distant from tumor. Samples were snap-frozen in liquid nitrogen and/or formalin-fixed, paraffin-embedded (FFPE) for histologic review, institutional tumor banking, and RNA extraction. Samples were stained with hematoxylin and eosin for assessment of cellularity and absence of tumor cells by a board-certified gynecologic pathologist.

### Molecular Analysis

RNA from FFPE (miRNeasy RNA FFPE Kit, Qiagen) and snap-frozen tissues (miRNeasy RNA mini Kit, Qiagen) was extracted per standard manufacturer protocols. Concentration and purity were measured using NanoDrop One Spectrophotometer (Thermo Fisher Scientific) and Qubit 3.0 Fluorometer (Life Technologies). For whole transcriptome library construction, 500 ng of total RNA for each sample was used. See Supplementary Data 2 for detailed methods.

### Endpoints

The primary endpoint of the trial was safety and survival outcomes, which was previously reported.^19^ In this secondary outcome analysis, renal toxicities and clinical outcomes were evaluated in two groups undergoing HIPEC with cisplatin: ST versus nST. Renal toxicity was defined as development of grade ≥ 1 AKI, chronic kidney disease (CKD), or proteinuria. Additional analyses included PFS by ST and nST use in primary and recurrent EOC patients. Exploratory analyses included correlation of toxicities with transcriptomic signature changes by whole transcriptomic sequencing (WTS) following HIPEC.

### Statistical Analysis

Baseline and postsurgical characteristics were compared between ST and nST patients by using the chi-square or Fisher’s exact test for categorical data, and the t-test (normally distributed) or Wilcoxon rank-sum (non-normally distributed) continuous variables. PFS was defined as the time in months from CRS and HIPEC to progression by CA-125 (Gynecologic Cancer Intergroup Criteria), imaging (CT or PET CT, per RECIST^20^), or clinical symptoms or deterioration. Kaplan-Meier and log-rank tests compared the PFS of patients with and without ST administration. Statistical analyses of differential RNAseq expression analysis of post-HIPEC samples are described in Supplementary Data 2.

## Results

### Clinical Characteristics

**Fig. 1:**
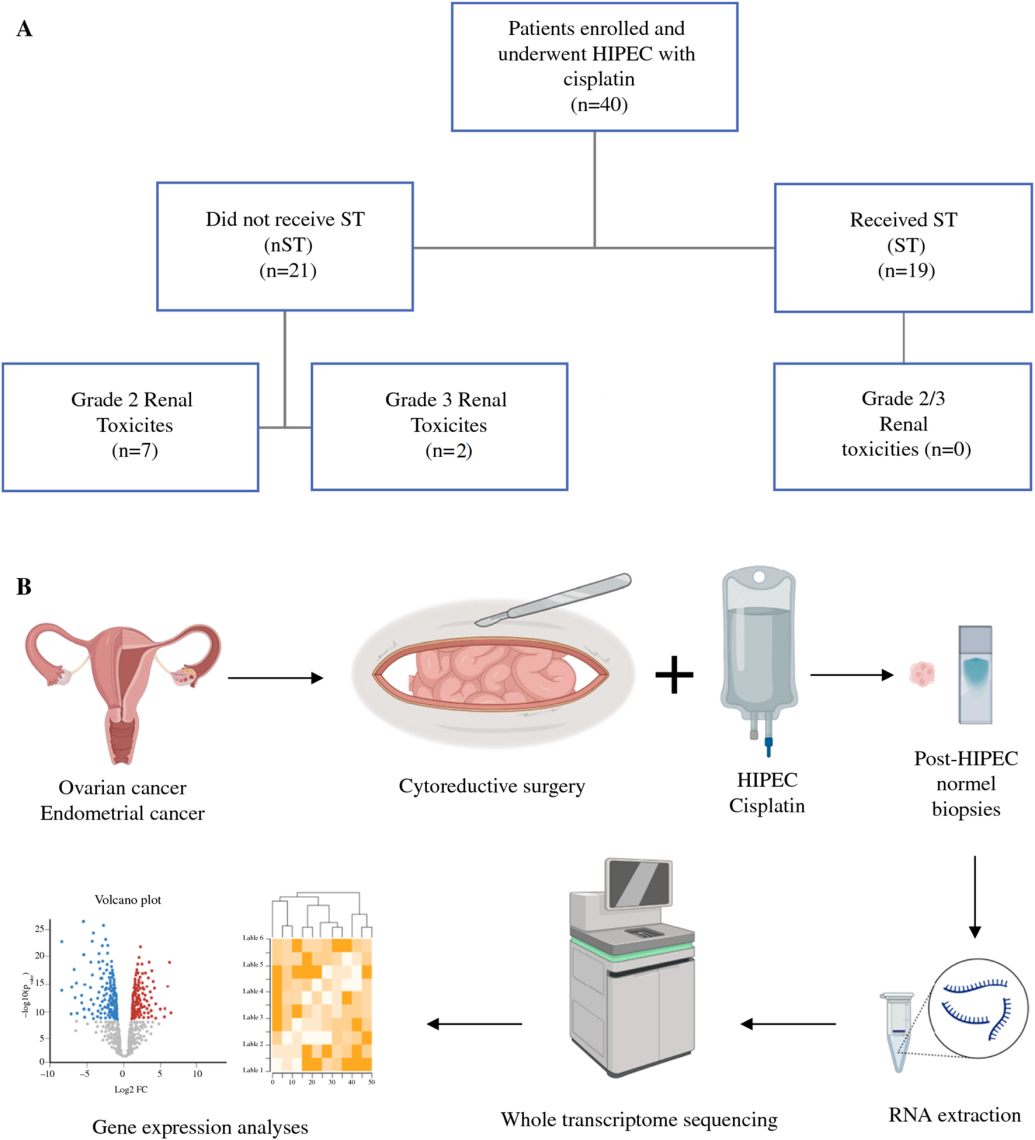
Schema for clinical and translational study components. **A** Sodium thiosulfate (ST) administration. **B** Translational transcriptomic study component

From 2014 to 2020, 60 patients were consented for the trial, and 40 of these patients were enrolled in the study comprising two groups: 21 who did not receive ST (nST, 2014–2018), and 19 who received ST (ST, 2019–2020). The study schema is shown in Fig. 1. The study enrolled 36 patients with EOC and 5 patients with EC. Half (20/40) of the patients had primary disease at the time of HIPEC, and half (20/40) were receiving HIPEC for recurrence. All patients had a pretreatment clinical FIGO stage of III (53%) or IV (47%). Baseline demographic and clinical characteristics are reported in Table 1. There were more recurrent cancer patients in the nST group compared with the ST group (61.9 vs. 36.8%, *p* = ns). The ST group had a lower median body mass index (BMI) compared with the nST group (23.4 vs. 28.5, *p* = 0.04). Other clinical characteristics between groups were similar, including age, high-grade serous histology, neoadjuvant chemotherapy exposure, history of preexisting hypertension, and presence of other medical comorbidities.

**Table 1:** Baseline demographic and clinical characteristics

Characteristic/demographic*	nST (*n* = 21)	ST (*n* = 19)	*p* value
Age at consent (y), median (IQR)	59.8 (52.7–66.9)	59.6 (46.6–66.3)	0.5
BMI, median (IQR)	28.5 (23.2–31.7)	23.4 (21.3–28.2)	0.04
Disease site and histology			
Ovarian and primary peritoneal	17 (81.0)	17 (89.4)	0.2
High grade serous	14 (67.0)	14 (73.7)	
Clear cell	2 (9.5)	**–**	
Low grade serous	**–**	2 (10.5)	
Mucinous	**–**	1 (5.3)	
Mixed	1 (4.8)	**–**	
Uterine	4 (19.0)	2 (10.5)	
Papillary serous carcinoma	1 (4.8)	1 (5.3)	
Adenocarcinoma NOS	1 (4.8)	1 (5.3)	
Carcinosarcoma	1 (4.8)	**–**	
Mixed	1 (4.8)	**–**	
Disease status			
Primary	8 (38.1)	12 (63.2)	0.1
Recurrent	13 (61.9)	7 (36.8)	
Length of stay (LOS), median (IQR)	9 (8–14)	8 (7–10)	0.3
Operating time (hours), median (IQR)	6.7 (6.0–8.7)	8.3 (7.1–11.8)	0.03
Operative PCI, median (IQR)	7 (5–11)	13 (7–18)	0.02
Days surgery to adjuvant chemotherapy, median (IQR)	58 (41–72)	41 (36–52)	0.06
PFS (months), median (95% CI)	14.2 (7.1, 22.4)	26.3 (26.6, NR)	0.01
OS (months), median (95% CI)	35.4 (24.1, 46.5)	NR (22.0, NR)	0.9
Follow-up (months), median (95% CI)	57.7 (32.8, NR)	27.7 (22.1, 31.8)	< 0.01
Type of debulking			
Consolidation	1 (4.8)	3 (15.8)	0.6
Interval	8 (38.1)	8 (42.1)	
Primary	1 (4.8)	1 (5.3)	
Secondary	10 (47.6)	7 (36.8)	
Tertiary	1 (4.8)	0 (0.0)	
HIPEC approach			
Closed	20 (95.2)	18 (94.7)	0.9
Minimally invasive	1 (4.8)	1 (5.3)	
Open	0 (0)	0 (0)	
Platinum sensitivity at recurrence			
Resistant	3 (23.1)	1 (14.3)	0.6
Sensitive	10 (76.9)	6 (85.7)	

Consolidation: HIPEC given following completion of first-line treatment; Interval: HIPEC and debulking following neoadjuvant chemotherapy followed by adjuvant chemotherapy; Primary: HIPEC and debulking performed prior to receipt of treatment; Secondary: HIPEC and debulking performed at first recurrence; Tertiary: HIPEC and debulking performed at second recurrence

*PCI* peritoneal carcinoma index, *PFS* progression-free survival, *OS* overall survival, *CI* confidence interval, *ST* sodium thiosulfate, *nST* no sodium thiosulfate, *HIPEC* hyperthermic intraperitoneal chemotherapy

*% Unless specified otherwise

### Surgical and Clinical Outcomes

Of the 40 enrolled patients, 38 (95%) underwent closed HIPEC technique, and two (5%) underwent laparoscopic technique. Twenty-eight of 40 (70%) patients underwent CCR0 resection, and 12 of 40 (30%) underwent CCR1 resection. Mean operative peritoneal carcinoma index (PCI) and median operative time were higher in the ST group compared with the nST group: 13 versus 7, *p* = 0.02 and 8.3 versus 6.7 h; *p* = 0.03, respectively. Median time to initiation of chemotherapy in the nST group was 58 (range 41–72) days compared with 41 (range 36–52) days in the ST group (*p* = 0.056). As the nST group preceded the ST group, median follow-up time was longer in the nST group (57.7 vs. 27.7 months, *p* < 0.01). Median LOS was similar between the two groups (nST 9 days vs. ST 8 days). Aside from renal failure and respiratory complications, postoperative complication rates based on Clavien-Dindo were similar between both groups (Table 2). There were 5/19 grade 2/3 respiratory-related Clavien-Dindo complications in the ST group that were likely secondary to fluid overload. No substantial changes in operating room staffing or anesthesia induction occurred during the 5-year study span. Changes were made to perioperative volume-status and base excess management as evidence emerged.^21,22^ Length of stay in the intensive care unit (ICU) is not reported given that admission at our institution may not accurately reflect of the severity of postoperative morbidity.

**Table 2:** Postoperative complications as scored using the Clavien-Dindo classification (20 of 40 patients had > 1 Clavien-Dindo complications recorded—11/21 nST and 9/19 ST patients)

Complication system	Description	(1) nST (*n* = 21)				(2) ST (*n* = 19)			
		I	II	IIIa	IVa	I	II	IIIa	IVa
Gastrointestinal	Uncontrolled abdominal pain post-operative						1		
	Postoperative ileus		1				1		
Hematologic	Anemia with or without transfusion required	1	3			2	4		
Infectious disease	Abdominal infection						1		
	Urinary tract infection		1						
	Pneumonia		1						
Renal/GU	Acute renal injury		8						
Respiratory	Pneumothorax						2	1	
	Respiratory failure				1		1		
	Pleural effusion					1	1		
Vascular	Absence of right distal radial pulse, cold extremity							1	
	Deep venous thrombosis		1						

Progression-free survival in the ovarian cancer subset of patients was similar between ST and nST patients (*p* = 0.6); median PFS months was 26.3 (95% confidence interval [CI] 6.8–33.9) vs. 14.3 (95% CI 7.2–26.9), respectively. Median overall survival was 35.4 months in the nST group (95% CI 25.1–NR) and was not reached in the ST group. Secondary analyses in primary and recurrent EOC groups did not show differences in survival between ST and nST patients (Supplementary Data 3). The percentage of patients who recurred was similar between ST and nST patients (73.7 vs. 71.4%).

### Adverse Event Monitoring

Table 3 reports a summary of all AE categories grade ≥ 2 reported in each group. Supplementary Table 1 details all reported AEs. No dose-limiting toxicities occurred in the cohort. All patients experienced at least one AE of grade ≥ 1. There were no grade 4 or 5 AEs in either group. Abdominal pain was the most common individual AE of any grade (Supplementary Table 1: nST 91%; ST 84%). Collectively, metabolism and nutrition disorders were the most common grade 3 AEs (Table 3: nST 14/40 (35.0%); ST 8/27 (29.6%)).

**Table 3:** Grade > 2* AEs attributable to surgery or cisplatin (possible, probable or definite); highest grade per event per patient

AE category	AE name	nST, *n* = 21		ST, *n* = 19	
		*n* (%)		*n* (%)	
		Grade 2	Grade 3	Grade 2	Grade 3
Blood and lymphatic system disorders	Anemia	5 (24)	10 (48)	6 (32)	7 (37)
Cardiac disorders	Atrial fibrillation			1 (5)	
	Sinus tachycardia			1 (5)	
	Supraventricular tachycardia	1 (5)			
	Ventricular tachycardia			1 (5)	
Gastrointestinal disorders	Abdominal pain	14 (67)		9 (47)	1 (5)
	Ascites				1 (5)
	Constipation	3 (14)		1 (5)	
	Diarrhea			1 (5)	
	Ileus	2 (10)	1 (5)	1 (5)	
	Nausea	5 (24)		2 (11)	
	Vomiting	1 (5)			
General disorders and administration site conditions	Edema face	1 (5)			
	Edema limbs	3 (14)		2 (11)	
	Fatigue	3 (14)		3 (16)	
Immune system disorders	Allergic reaction			1 (5)	
Infections and infestations	Abdominal infection		1 (5)		1 (5)
	Lung infection	1 (5)			
	Urinary tract infection	1 (5)			
Investigations	Alanine aminotransferase increased	3 (14)	1 (5)		2 (11)
	Aspartate aminotransferase increased	1 (5)	4 (19)		2 (11)
	Blood bilirubin increased	1 (5)			
	Creatinine increased	5 (24)	2 (10)		
	Lymphocyte count decreased	1 (5)		2 (11)	
	Platelet count decreased	1 (5)	1 (5)	3 (16)	
	Weight gain				1 (5)
Metabolism and nutrition disorders	Acidosis		5 (26)		
	Alkalosis		1 (5)		
	Anorexia	1 (5)			
	Hyperglycemia	2 (10)			
	Hypernatremia	1 (5)			
	Hypoalbuminemia	8 (38)	1 (5)	7 (37)	
	Hypocalcemia	7 (33)	1 (5)	2 (11)	1 (5)
	Hypokalemia	6 (29)	1 (5)	1 (5)	2 (11)
	Hypomagnesemia		1 (5)	1 (5)	3 (16)
	Hyponatremia		2 (10)		
	Hypophosphatemia	2 (10)	2 (10)	2 (11)	2 (11)
Musculoskeletal and connective tissue disorders	Pain in extremity			1 (5)	
Nervous system disorders	Lethargy	1 (5)			
	Nystagmus	1 (5)			
	Peripheral motor neuropathy			1 (5)	
Psychiatric disorders	Anxiety	1 (5)		2 (11)	
	Confusion			1 (5)	
	Hallucinations	1 (5)		2 (11)	
Renal and urinary disorders*	Acute kidney injury	4 (19)	2 (10)		
	Chronic kidney disease		1 (5)		
	Proteinuria	1 (5)			
	Urinary frequency	1 (5)			
	Urinary incontinence			1 (5)	
	Urinary retention			1 (5)	
Respiratory, thoracic and mediastinal disorders	Dyspnea	2 (10)		3 (16)	
	Hypoxia	2 (10)	1 (5)	2 (11)	1 (5)
	Pleural effusion		1 (5)	4 (21)	1 (5)
	Pleural hemorrhage			1 (5)	
	Pneumothorax			2 (11)	1 (5)
	Pulmonary edema	1 (5)			
	Sore throat			1 (5)	
Vascular disorders	Hypertension	3 (14)		3 (16)	
	Hypotension	2 (10)			
	Thromboembolic event		1 (5)		
	Cold hand				1 (5)

*AE* adverse event, *ST* sodium thiosulfate, *nST* no sodium thiosulfate

*Grade 1 events noted within the renal and urinary disorders indicated one occurrence of proteinuria in the nST group. 21/21 (100%) of nST patients and 18/19 (94.7%) of ST patients incurred any Grade 2 or 3 toxicity. There is no significant difference in this overall comparison between the two groups (*p* = 0.3)

### Renal Toxicities

**Fig. 2:**
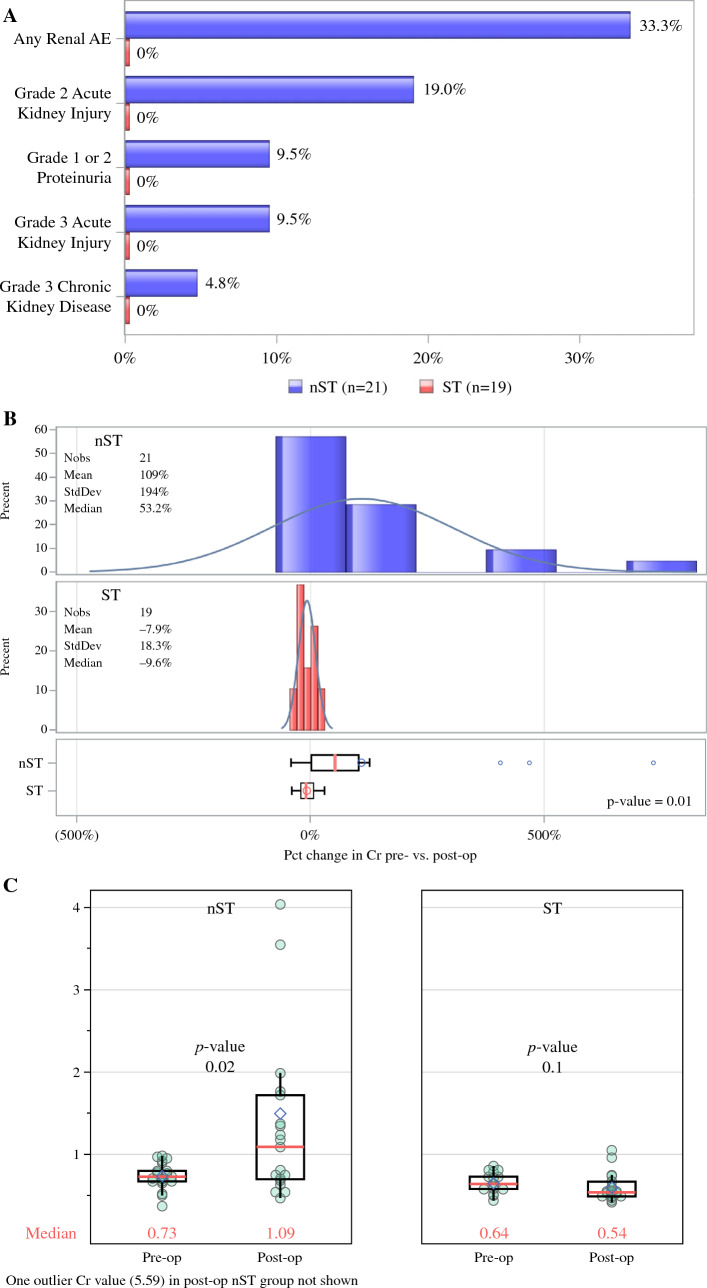
Renal adverse events (AEs) and creatinine changes by ST exposure. **A** Renal AEs and grade 2 and 3 acute kidney injury and chronic kidney disease events for the nST and ST arms. **B** Distribution of percent change in creatinine by ST exposure. **C** Differences between preoperative and postoperative creatinine values for the nST and ST arms

There were no grade 2 or 3 renal AEs in the ST group (Fig. 2A). A total of nine renal AEs were seen in seven of 21 (33.3%) patients in the nST group; one of 21 (4.8%) developed a grade 1, four of 21 (19.0%) patients developed grade 2 renal AEs, and two of 21 (9.5%) developed grade 3 renal AEs. Of those in the nST group who developed grade 3 renal AEs, two had AKI (9.5%) and one developed CKD (4.8%). Four patients were identified with hypertension at baseline. Three of these patients experienced nephrotoxicity (nST), and one did not (ST).

Preoperative and postoperative Cr values were obtained and compared for patients in both groups. There was no statistically significant difference between preoperative and postoperative Cr among patients who received ST (median preoperative vs. postoperative Cr, 0.64 vs. 0.54, *p* = 0.097). Percent change between preoperative and postoperative Cr was significantly higher in the nST group (median + 53%) versus ST group (median − 9.6%, *p* = 0.003; Fig. 2B). Median preoperative and postoperative Cr in the nST group were 0.73 and 1.09, respectively (*p* = 0.02; Fig. 2C). When percent change between preoperative and postoperative Cr was compared, the nST group had a higher proportion of patients with a net increase (Supplementary Data 4A). There was a statistically significant broader range of postoperative Cr values in the nST arm compared with the ST arm (*p* = 0.0001; Supplementary Data 4B).

### Toxicity-Related Gene and Mutational Signatures

**Fig. 3:**
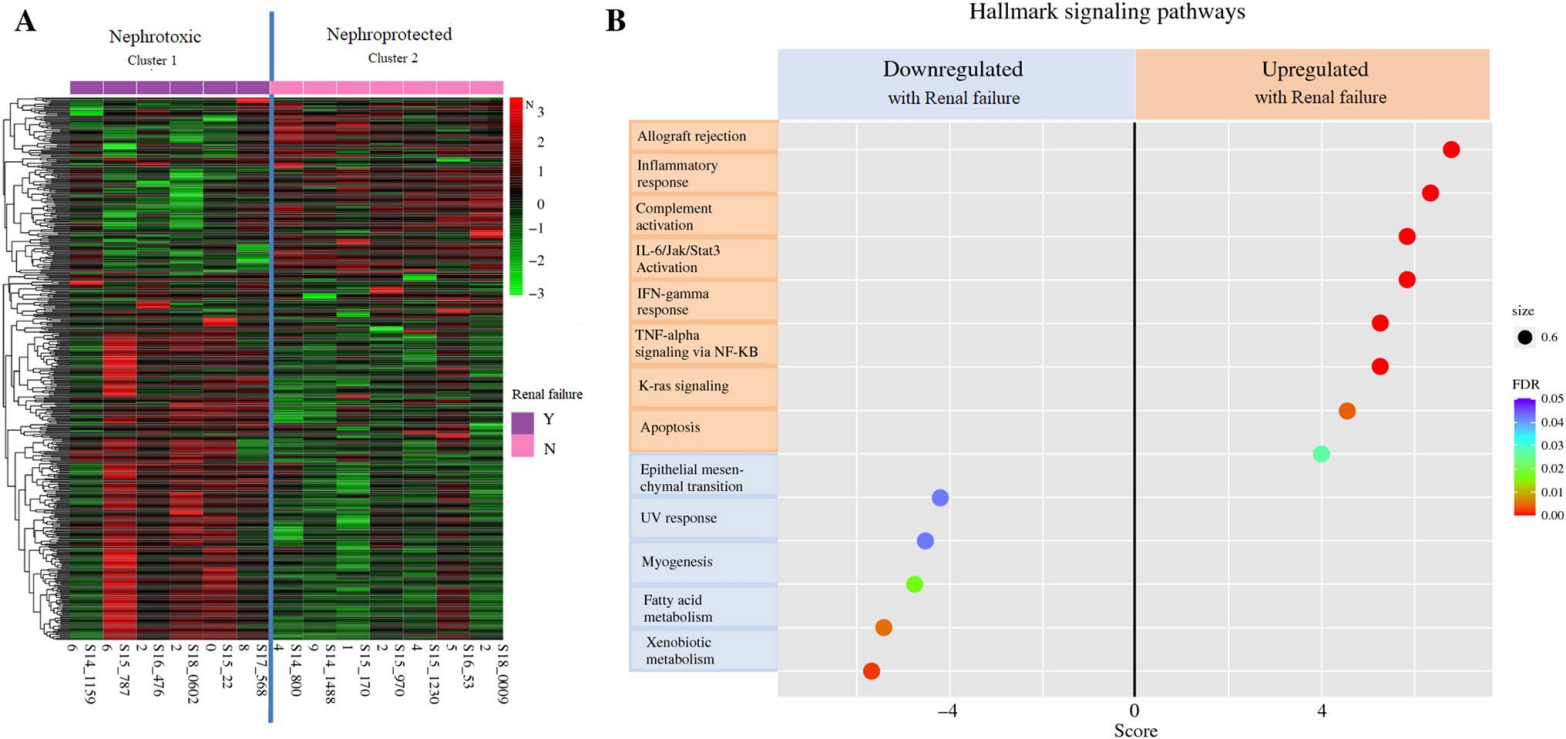
WTS data for the nST group. **A** Genomic heatmap showing the difference in genetic clusters between the nephrotoxic (NT) and nephroprotected (NP) groups. **B** Hallmark signaling pathway analysis table illustrating the global molecular processes up and downregulated in the NT group

Whole transcriptomic sequencing identified correlations between renal AEs and gene expression in normal samples from 13 patients in the nST group with available WTS (Fig. 3). Patients were divided into two groups: nephrotoxic (NT) and nephroprotected (NP). Post-HIPEC peritoneal sampling locations were similar between both groups (Supplementary Table 2). Clinical characteristics were similar between the two analyzed groups (Supplementary Table 3). Patients in the NT group were defined as those who had a postoperative Cr rise (*n* = 6). Patients in the NP group were those without a postoperative Cr rise (*n* = 7). Supervised clustering analyses identified distinct WTS signatures for NT and NP patients. There was a notable difference in genetic clusters (Fig. 3A) between the NT and NP groups. Analysis of hallmark signaling metabolic pathways modulated in the NT group demonstrated upregulation in inflammation and immune-mediated signaling and downregulation in metabolic and catabolic processes (Fig. 3B). GO BIO signaling pathway analysis showed that most upregulated genes in the NT group are involved in immune function and inflammation, whereas most downregulated genes are part of metabolic pathways (Supplementary Data 5A). Supplementary Data 5B shows the specific upregulated and downregulated genes in the NT group, which show a similar trend.

## Discussion

Despite enthusiasm for HIPEC in EOC, routine use of HIPEC is marred by its toxicities and skepticism over its true clinical benefit. Despite a well-established incidence of nephrotoxicity from HIPEC with cisplatin, management and prevention of this toxicity vary widely. The Peritoneal Surface Oncology Group International (PSOGI) recently performed a consensus among international HIPEC experts to derive recommendations regarding the indications and delivery of HIPEC regimens.^5^ While the evidence in favor of using nephroprotection in HIPEC with cisplatin was low, the consensus group recommended the routine use of nephroprotection in all patients undergoing HIPEC with cisplatin. Additionally, ST was considered the preferred drug for nephroprotection by most experts.

Despite preferred expert opinions, there have been limited studies investigating HIPEC-specific nephrotoxicities. To date, retrospective studies have compared the nephroprotective effects of ST versus no ST when given in conjunction with HIPEC with cisplatin.^23^ While ST was used in all patients enrolled in OVHIPEC-1 without significant renal toxicities, ST use versus no ST use has not been evaluated in a prospective manner. We present an ancillary study that compares use of ST as a nephroprotectant versus no nephroprotectant use in patients undergoing HIPEC with cisplatin. While a theoretical concern exists that ST may abrogate cisplatin efficacy given its known anti-alkylating properties, we demonstrated no difference in PFS in patients with gynecologic malignancies who received ST (versus nST) for renal prophylaxis.^11^

A major strength of our study is the evaluation of ST use versus no ST use on renal function as a primary outcome. Although some trials have shown the nephroprotective effect of ST, they have been largely retrospective or observational in design.^23,24^ The OVHIPEC-1 study demonstrated improved survival outcomes in advanced stage ovarian cancer patients and included ST prophylaxis in the protocol, and no renal AEs were reported.^1^ Baseline creatinine was similar between the ST and nST groups. Our trial demonstrated the safety, feasibility, and efficacy of HIPEC with cisplatin with ST in primary and recurrent EOC and EC patients for renal protection. No grade 4 or 5 AEs were reported, and the most common grade 3 AEs experienced (anemia; metabolic and electrolyte disturbances) were most often associated with CRS. Renal AEs and significant differences in postoperative Cr were only experienced by those who did not receive ST. These findings are consistent with OVHIPEC-1, where no significant renal toxicities were reported with the routine use of ST.^1^ Median OR time was slightly shorter in patients with nephrotoxicity (6.4 vs. 7.5 h) compared with those without. Even with these increased operative times, the ST group did not have renal AEs. Our data reiterate support for the standard use of ST for nephroprotection in patients undergoing HIPEC with cisplatin.

While the median days to initiation of adjuvant chemotherapy was not statistically significant in our study, a trend favored the ST group in initiating adjuvant chemotherapy sooner than the nST group. There also was a trend toward improved median PFS amongst the ovarian cancer patients analyzed in the ST group compared with the nST group, although this may be due to imbalance of primary EOC predominance in the ST group compared with recurrent EOC predominance in the nST group. In the subcohorts of primary EOC and recurrent EOC, there was no difference in PFS between the nST and ST groups. We sought homogeneity for survival analysis and thus excluded patients with uterine cancer. The uterine cancer cohort was small (*n* = 6) and imbalanced toward recurrent disease.

We performed transcriptomic analyses in normal peritoneal samples to identify molecular signatures that correlate with HIPEC-induced renal toxicities. These novel analyses investigate what is occurring at a transcriptional level in patients who experience renal toxicity from HIPEC with cisplatin. Gene pathways related to immune modulation were significantly upregulated in patients who experienced renal failure, many of which are related to neutrophil, T-cell, and B-cell activation.

The upregulation of inflammatory cells, such as neutrophils and T cells, suggests an acute inflammatory response resulting in AKI. Indeed, immune and inflammatory mechanisms have been recognized as important mediators of cisplatin nephrotoxicity.^6^ While immune mediated mechanisms of nephrotoxicity from intravenous cisplatin have been recognized, this is the first observation in IP cisplatin administration in humans, to our knowledge. Our inclusion of peritoneal transcriptomics in patients without nephrotoxicity demonstrate a strikingly differently patterned genetic heatmap compared with those with nephrotoxicity. Conversely, downregulated pathways were related to metabolism, pointing to a reduced metabolism phenotype in patients at risk for renal toxicities. Identifying potential biomarkers associated with nephrotoxicity may help to optimize HIPEC with cisplatin by identifying patients who are not ideal candidates for HIPEC or who may require additional supportive therapy to undergo HIPEC. Although kidney biopsies were not obtained in this study, there is evidence that cisplatin-related nephrotoxicity is induced by cytokine-mediated inflammation.^6^ The microenvironment in the peritoneum may reflect a systemic proinflammatory state given that is where absorption of HIPEC with cisplatin is directly occurring.

Our study is limited by the lack of randomization, which led to the nST group preceding the ST group with resultant median follow up differences. ST administration was initiated after interim analysis revealing significant renal toxicities. This limited our ability to exclude possible confounders in our subject selection. This chronological recruitment for both groups accounts for the difference in median follow-up between the nST and ST groups (57.7 vs. 27.7 months), given that nST subjects were recruited first. An additional limitation includes a slight imbalance in disease characteristics between the nST and ST groups. The nST group had more recurrent cancer cases, whereas the ST group had more primary cases. Although it could be assumed that more heavily pretreated, recurrent EOC patients may have worse renal function, baseline Cr between the two groups was similar. Mean operating time and PCI were lower in the nST group. This could be associated with decreased surgical complexity and associated complications in the nST group, thus reducing the likelihood for acute renal failure in this group. BMIs were significantly different between the two groups (ST: 23.4 vs. nST: 28.5, *p* = 0.04), but within the nST group, patients who had renal toxicity versus no renal toxicity had no significant difference in BMI. The median BMI for patients with and without observed renal AEs was 27.6 and 28.8, respectively (*p* value from Wilcoxon rank-sum test was 0.6) within the nST group. Even though the patient group with renal AEs was small (*n* = 7), the results do not lead us to believe that BMI was an important factor in the incidence of AEs among the nST patients. Patients in the ST group had more postoperative complications secondary to fluid overload. Given that the ST patients underwent HIPEC treatment chronologically later than their nST counterparts, perioperative volume management adjustments were made as recommendations emerged in the literature.^21,22^ Cisplatin dosage in our study was different than the OVHIPEC-1 study; 75 versus 100 mg/m^2^, respectively. However, while 100 mg/m^2^ is the currently accepted dose for HIPEC with cisplatin in the first line setting for ovarian cancer,^25^ other studies, including the Korean^13^ and CARCINO-HIPEC^26^ trials used 75 mg/m^2^. Lastly, although our study population was primarily comprised of EOC patients, there was some heterogeneity with the inclusion of six patients (15%) with uterine cancer. However, given that our primary endpoint was renal AEs secondary to administration of HIPEC with cisplatin, it is unlikely that the variation in histology or disease site present any confounding factors.

## Conclusion

We contribute to the growing pool of literature showing that ST abrogates nephrotoxicities induced by HIPEC with cisplatin and does not result in reduced PFS. The lack of renal toxicities associated with ST use and its stability in serum Cr, in contrast to the significant increase in serum Cr in patients without ST use, underscore the importance nephroprotection in HIPEC with cisplatin for patients with ovarian cancer.

### Supplementary Information

Below is the link to the electronic supplementary material.

Supplementary file1 (PDF 669 kb)
