# Supplementary material for: Impact of Sodium Thiosulfate on Prevention of Nephrotoxicities in HIPEC: An Ancillary Evaluation of Cisplatin-Induced Toxicities in Ovarian Cancer
Source: Ann Surg Oncol. 2023 Sep 14;30(13):8144–55. doi: 10.1245/s10434-023-14216-6 (PMC10625947; doi:10.1245/s10434-023-14216-6)
Supplement: Supplementary file 1 — Supplementary file1 (PDF 669 kb) [file 10434_2023_14216_MOESM1_ESM.pdf]

Supplementary Data 1: Eligibility, inclusion, and exclusion criteria

Eligibility criteria

Patients must satisfy all of the following conditions in order to be eligible for study enrollment and participation. Eligible patients may be deemed ineligible based on intra-operative findings or conditions that cannot be evaluated or predicted pre-operatively.

Inclusion Criteria

- 18 years of age or older and provided informed consent.
- Patient with primary FIGO Stage III or IV, or recurrent ovarian, fallopian tube, peritoneal carcinoma, or uterine cancer, confined to abdominal cavity, including those who have completed neoadjuvant chemotherapy and primary surgery.
- GOG or ECOG performance status ≤1, or Karnofsky scale (KPS) ≤70%.
- Patients who are platinum-sensitive or platinum resistant.
- Candidate for potentially radical, maximal effort cytoreductive surgery at the discretion and expertise of the treating physician.
- For patients with newly diagnosed –ovarian/tubal/peritoneal cancer who have received pre-operative neoadjuvant chemotherapy, evidence of response must be documented by at least one of the following:
    - Decline in serum CA125 level.
    - At least a 30% decrease in the sum of the longest diameter of target lesions on radiographic imaging.
    - Improvement of ascites volume.
    - Neoadjuvant chemotherapy must be held for at least 3 weeks prior to surgery.

    - Resolution of any effects of prior therapy (except alopecia and peripheral neuropathy) to the current NCI Common Terminology Criteria for Adverse Events (NCI CTCAE) grade ≤1 and to baseline laboratory values as defined below.

- Patients must have adequate:
    - Bone marrow function: HGB ≥9g/dL, WBC ≥3,000/mcL, ANC ≥1,500/mcL.
    - PLT ≥100,000/mcL.
    - Hepatic function: Total bilirubin within normal institutional limits, SGOT/SGPT <2.5 x institutional upper limit of normal (ULN).
    - Renal function: Creatinine <1.5 x ULN or creatinine clearance >60ml/min according to Cockroft-Gault formula.
    - Neurologic function: Neuropathy (sensory and motor) NCI CTCAE grade ≤2.
    - Blood coagulation parameters: PT such that international normalized ratio (INR) is <1.5 (or an in-range INR, usually between 2 and 3, if a patient is on a stable dose of therapeutic warfarin or low molecular weight heparin) and a PTT <1.2 times control.
    - Serum albumin ≥2.5.
- No active infection requiring antibiotics.
- Preoperative or intraoperative (frozen section) diagnosis of ovarian, peritoneal, fallopian tubal or uterine cancer.
- Surgery achieves either no gross residual disease (R0) or optimal cytoreductive status defined as no single lesion measuring more than 5.0mm in its greatest diameter.
- Stable from a cardiopulmonary standpoint to continue with prolonged surgery and anesthesia.

Exclusion Criteria

- Patients with active extra-abdominal disease including active malignant pleural effusion. Patients who have been successfully treated with neoadjuvant chemotherapy and no longer have (malignant) pleural effusions may be included.
- Patients whose disease has progressed following at least 3 cycles of neoadjuvant chemotherapy as defined by at least one of the following:
  - Doubling of serum CA-125 level.
  - At least a 20% increase in the sum of the longest diameter of target lesions, taking as reference the smallest sum longest diameter recorded since the treatment started or the appearance of one or more new lesions..
  - Clinical deterioration (worsening ascites, carcinomatous ileus, malignant bowel obstruction, severe hypoalbuminemia, declining performance status).
- Cardiac or pulmonary conditions that preclude aggressive cytoreductive surgery.
- Patients whose circumstances do not permit completion of the study or the required follow-up.
- Pregnant, nursing, or of childbearing potential and refuse hysterectomy or bilateral salpingo-oophorectomy.
- Other active invasive malignancies, with the exception of non-melanoma skin cancer and breast cancer (if without evidence of disease 1 year after completion of treatment).
- Metastatic non-gynecologic or breast primaries.
- Sub-optimal resection as their surgical outcome.
- Intraoperative frozen section suggesting hepatobiliary, pancreatic, adrenal, or urinary tract cancer.

Supplementary Data 2

Methodology

Next generation sequencing

Libraries were constructed using KAPA Stranded RNA-Seq Kit with Riboerase (Roche) following manufacturer's instructions. Samples were heat fragmented according to their Agilent Tapestation RIN and DV200 values following manufacturer's instructions. Final libraries were assessed on the Agilent Tapestation 4200 and quantified using the Qubit fluorometer (Thermo Fisher Scientific) and equimolarly pooled. Final library pools were sequenced using Illumina iSeq using Illumina's iSeq 100 i1 Reagent Kit (Illumina) to verify pool breakdowns.

Differential gene expression analysis

A filter cutoff of fold-change ≥±2 and P value of <0.05 identified differential gene expression changes based on QLF-testing(edgeR). GSEA pre-ranked software computed normalized enrichment scores (NES) and false discovery rate (FDR) values for Kegg gene sets, downloaded from MSigDBv7.2. [1] Genes were pre-ranked based on log fold-change and P values. Significantly changed signaling pathways (FDR <0.05) were depicted as a bubble plot. Average of fold-change value of genes in each pathway was calculated and expressed as a bubble size.

Library pools with a 1% phiX spike-in were clustered and sequenced by synthesis using Illumina's NovaSeq 6000 S1 (200 cycles) kit on the Illumina NovaSeq 6000 for paired 100bp reads (Illumina). 60 million total reads were targeted for each RNA sample.

Reads from FASTQ files were mapped against hg19 human reference genome using STAR v2.7.4. [2] Raw read counts mapped to genes were measured using the BAM format file by feature Counts in R subread R package v1.34.7. [3] 13,748 coding genes were analyzed for transcript abundance and poorly expressed genes were eliminated based on the criteria of RPKM <1 for at least 2 samples. Read counts were normalized using the Trimmed Mean of M-values normalization method.

References

1. Liberzon A, Birger C, Thorvaldsdottir H et al. The Molecular Signatures Database (MSigDB) hallmark gene set collection. Cell Syst 2015; 1: 417-425.

2. Dobin A, Davis CA, Schlesinger F et al. STAR: ultrafast universal RNA-seq aligner. Bioinformatics 2013; 29: 15-21.

3. Liao Y, Smyth GK, Shi W. The R package Rsubread is easier, faster, cheaper and better for alignment and quantification of RNA sequencing reads. Nucleic Acids Res 2019; 47: e47.

4. Mayakonda A, Lin DC, Assenov Y et al. Maftools: efficient and comprehensive analysis of somatic variants in cancer. Genome Res 2018; 28: 1747-1756.

5. Rosenthal R, McGranahan N, Herrero J et al. DeconstructSigs: delineating mutational processes in single tumors distinguishes DNA repair deficiencies and patterns of carcinoma evolution. Genome Biol 2016; 17: 31.

6. Alexandrov LB, Kim J, Haradhvala NJ et al. The repertoire of mutational signatures in human cancer. Nature 2020; 578: 94-101.

7. Yachida N, Yoshihara K, Suda K et al. Biological significance of KRAS mutant allele expression in ovarian endometriosis. Cancer Sci 2021; 112: 2020-2032.

Supplementary Data 3: Survival curves for ovarian cancer patients in ST v. nST groups

A. Progression-free survival in all ovarian patients, by ST

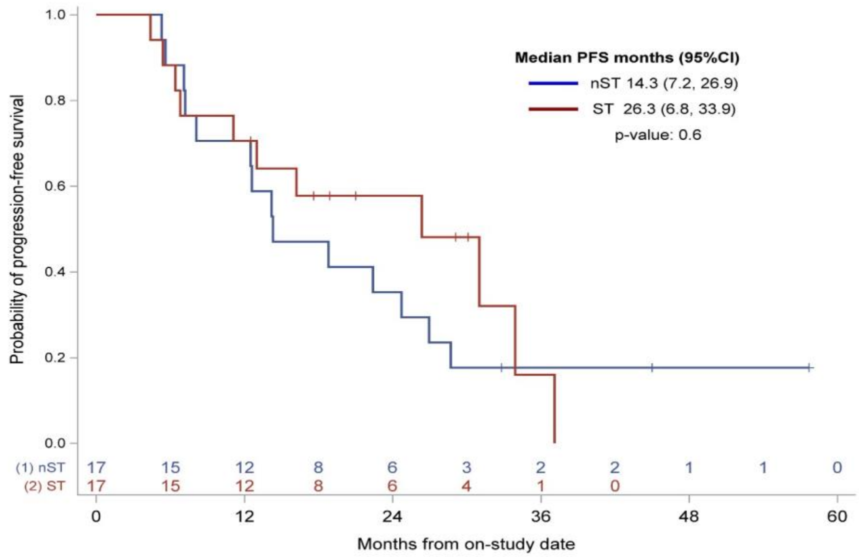

B. Progression-free survival in primary ovarian patients, by ST

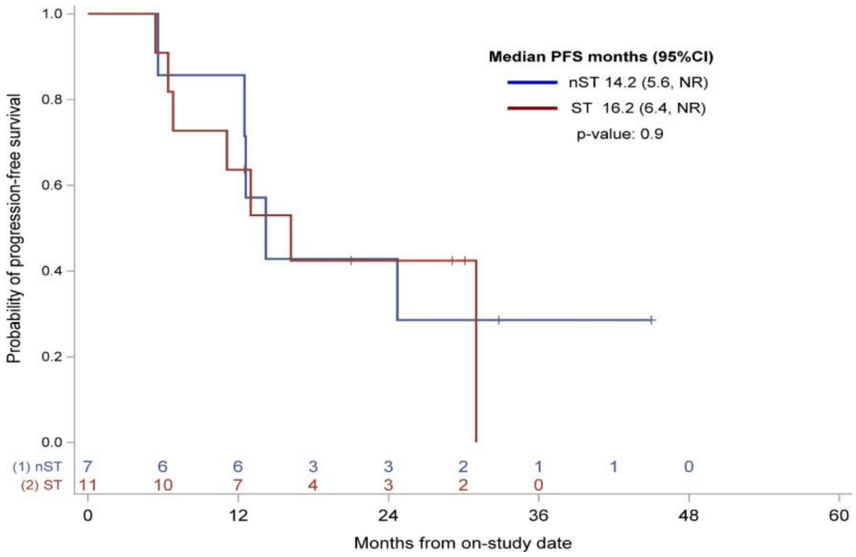

C. Progression-free survival in recurrent ovarian patients, by ST

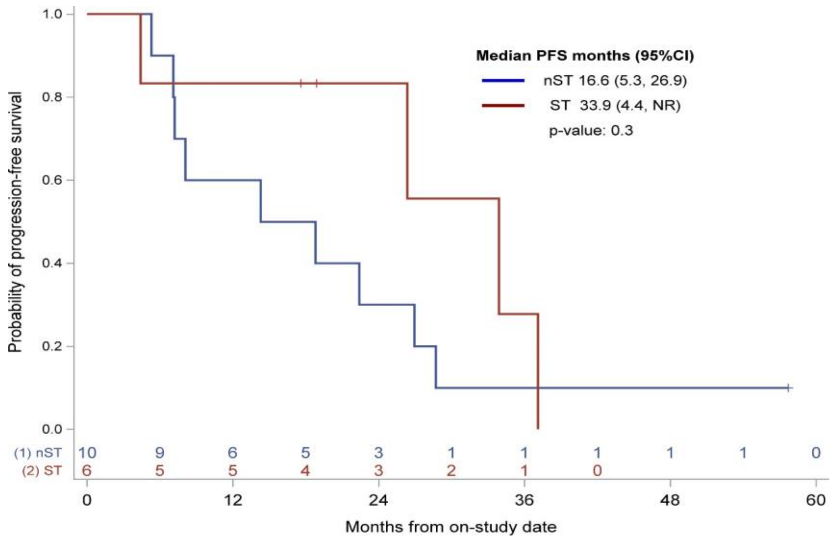

Supplementary Table 1: Grade ≥1 AEs attributable to surgery or cisplatin (possible, probable or definite); highest grade per event per patient

AE Category	AE Name	nST, n=21 n (%)			ST, n=19 n (%)		
		G1	G2	G3	G1	G2	G3
**Blood and lymphatic system disorders**	Anemia	1 (5)	5 (24)	10 (48)	2 (11)	6 (32)	7 (37)
**Cardiac disorders**	Atrial fibrillation					1 (5)	
	Sinus tachycardia	4 (19)			9 (47)	1 (5)	
	Supraventricular tachycardia		1 (5)				
	Ventricular tachycardia					1 (5)	
**Gastrointestinal disorders**	Abdominal distension	1 (5)			2 (11)		
	Abdominal pain	5 (24)	14 (67)		6 (32)	9 (47)	1 (5)
	Ascites						1 (5)
	Bloating				2 (11)		
	Constipation		3 (14)			1 (5)	
	Diarrhea	3 (14)			3 (16)	1 (5)	
	Dry mouth	1 (5)					
	Fecal incontinence				1 (5)		
	Flatulence	1 (5)					
	Ileus		2 (10)	1 (5)		1 (5)	
	Nausea	11 (52)	5 (24)		8 (42)	2 (11)	
	Vomiting	5 (24)	1 (5)		4 (21)		
**General disorders and administration site conditions**	Edema face		1 (5)				
	Edema limbs	6 (29)	3 (14)		3 (16)	2 (11)	
	Fatigue	7 (33)	3 (14)		7 (37)	3 (16)	
	Fever	4 (19)			1 (5)		
	Pain	1 (5)					
**Immune system disorders**	Allergic reaction					1 (5)	
**Infections and infestations**	Abdominal infection			1 (5)			1 (5)
	Lung infection		1 (5)				
	Urinary tract infection		1 (5)				
**Injury, poisoning and procedural complications**	Bruising				1 (5)		
**Investigations**	Alanine aminotransferase increased	6 (29)	3 (14)	1 (5)	8 (42)		2 (11)
	Alkaline phosphatase increased	6 (29)			5 (26)		
	Aspartate aminotransferase increased	6 (29)	1 (5)	4 (19)	8 (42)		2 (11)
	Blood bilirubin increased	5 (24)	1 (5)				
	Creatinine increased	2 (10)	5 (24)	2 (10)			
	Lymphocyte count decreased		1 (5)			2 (11)	
	Platelet count decreased	5 (24)	1 (5)	1 (5)	4 (21)	3 (16)	
	Weight gain						1 (5)
	White blood cell decreased	1 (5)					

AE Category	AE Name	nST, n=21<br>n (%)			ST, n=19<br>n (%)		
		G1	G2	G3	G1	G2	G3
**Metabolism and nutrition disorders**	Acidosis	2 (10)		5 (24)			
	Alkalosis			1 (5)			
	Anorexia	1 (5)	1 (5)		2 (11)		
	Hyperglycemia	6 (29)	2 (10)				
	Hypermagnesemia	3 (14)			1 (5)		
	Hypernatremia		1 (5)		1 (5)		
	Hypertriglyceridemia	2 (10)					
	Hypoalbuminemia	3 (14)	8 (38)	1 (5)	4 (21)	7 (37)	
	Hypocalcemia	8 (38)	7 (33)	1 (5)	11 (58)	2 (11)	1 (5)
	Hypokalemia	5 (24)	6 (29)	1 (5)	6 (32)	1 (5)	2 (11)
	Hypomagnesemia	11 (52)		1 (5)	6 (32)	1 (5)	3 (16)
	Hyponatremia	12 (57)		2 (10)	8 (42)		
	Hypophosphatemia	3 (14)	2 (10)	2 (10)	5 (26)	2 (11)	2 (11)
**Musculoskeletal and connective tissue disorders**	Arthralgia	1 (5)					
	Generalized muscle weakness				7 (37)		
	Muscle weakness lower limb	1 (5)			1 (5)		
	Myalgia	1 (5)					
	Pain in extremity				3 (16)	1 (5)	
**Nervous system disorders**	Dizziness	2 (10)			6 (32)		
	Headache	1 (5)					
	Lethargy		1 (5)				
	Nystagmus		1 (5)				
	Peripheral motor neuropathy					1 (5)	
	Peripheral sensory neuropathy	1 (5)					
	Somnolence	1 (5)					
**Psychiatric disorders**	Agitation	1 (5)			1 (5)		
	Anxiety		1 (5)		3 (16)	2 (11)	
	Confusion	1 (5)				1 (5)	
	Hallucinations	2 (10)	1 (5)			2 (11)	
	Insomnia	2 (10)			1 (5)		
**Renal and urinary disorders**	Acute kidney injury		4 (19)	2 (10)			
	Chronic kidney disease			1 (5)			
	Proteinuria	1 (5)	1 (5)				
	Urinary frequency		1 (5)				
	Urinary incontinence					1 (5)	
	Urinary retention					1 (5)	

AE: Adverse event; ST: sodium thiosulfate; nST: no sodium thiosulfate

**Respiratory, thoracic and mediastinal disorders**	Cough	2 (10)					
	Dyspnea	3 (14)	2 (10)			3 (16)	
	Hiccups	1 (5)					
	Hypoxia		2 (10)	1 (5)		2 (11)	1 (5)
	Pharyngolaryngeal pain	1 (5)					
	Pleural effusion			1 (5)		4 (21)	1 (5)
	Pleural hemorrhage					1 (5)	
	Pneumothorax					2 (11)	1 (5)
	Productive cough	1 (5)					
	Pulmonary edema		1 (5)				
	Sore throat	3 (14)			2 (11)	1 (5)	
**Skin and subcutaneous tissue disorders**	Rash maculo-papular				1 (5)		
**Vascular disorders**	Hypertension		3 (14)		1 (5)	3 (16)	
	Hypotension	3 (14)	2 (10)				
	Thromboembolic event			1 (5)			
	cold hand						1 (5)

Supplementary Data 4: Distribution of postoperative creatinine values for ST and nST groups

A.

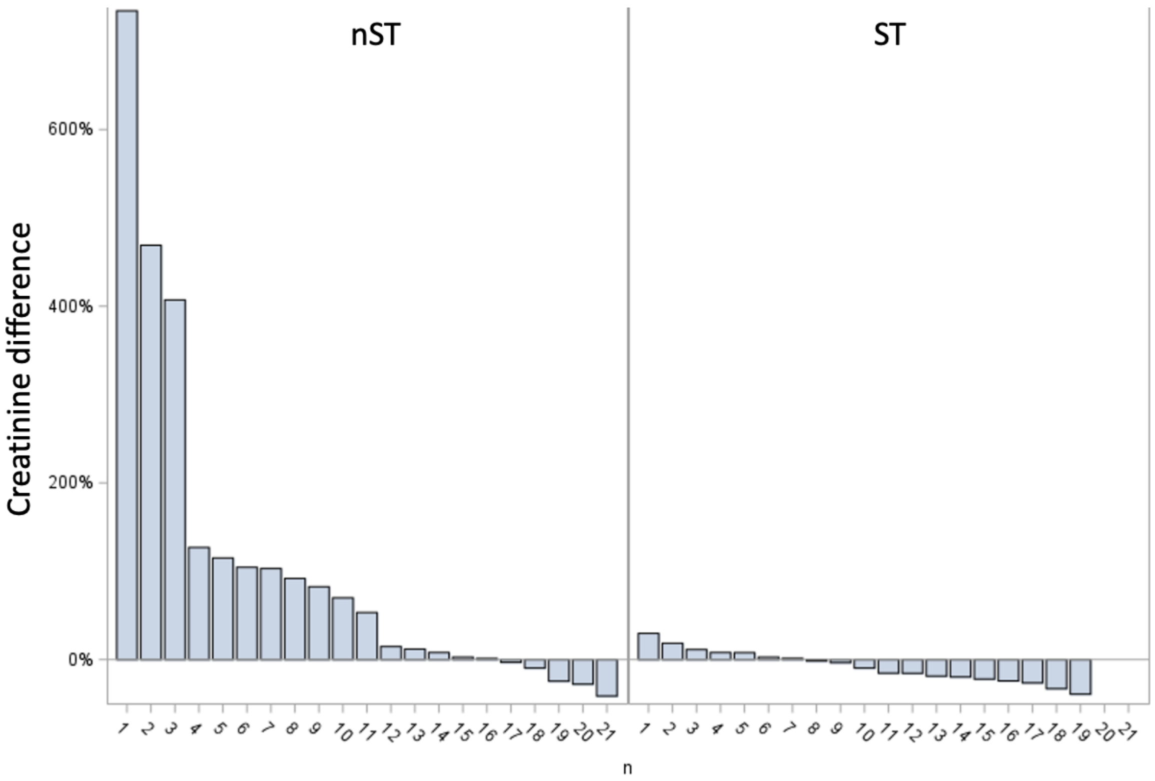

B.

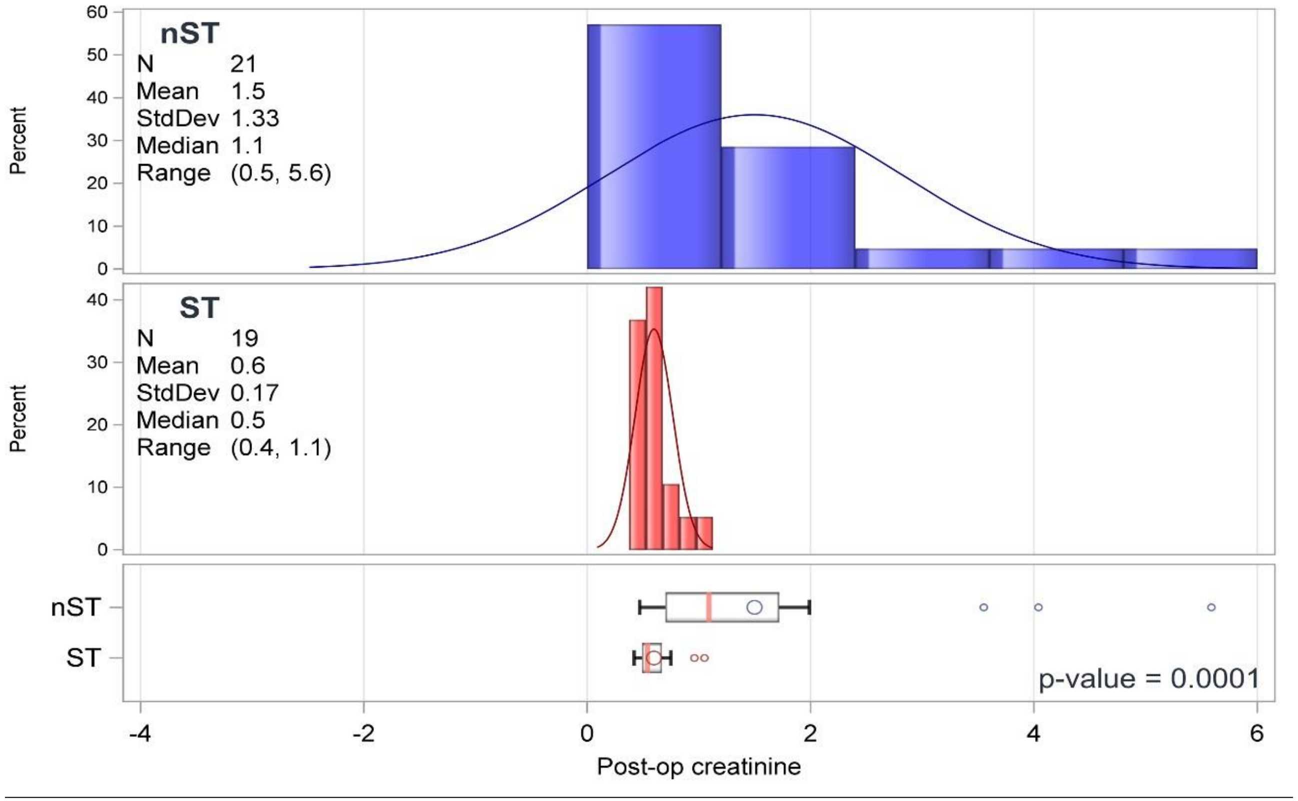

Supplemental Table 2: Location of post-HIPEC peritoneal sampling for transcriptomic sequencing in A) nephrotoxicity protected (NTP) and B) nephrotoxic (NTT) groups.

A.

Subject ID	Location
S18_00092	Right diaphragm
S16_535	Omentum
S15_1701	Liver capsule
S15_12304	Left pelvic sidewall
S15_9702	Omentum
S14_8004	Omentum
S14_14889	Abdominal wall

B.

Subject ID	Location
S15_220	Abdominal wall
S17-5688	Right ovary
S18_06022	Colon
S15_7876	Liver capsule
S16_4762	Right diaphragm
S14_11596	Abdominal wall

Supplementary Table 3: Clinical characteristics between nephrotoxicity protected (NTP) and nephrotoxicity (NTT) groups who underwent transcriptomic analysis.

	NTP (n=7)	NTT (n=6)	p-value
nST v. ST			
nST	7 (100.0%)	6 (100.0%)	NA
ST	0 (0.0%)	0 (0.0%)	
Median age at consent (years), (IQR)	58 (52 - 60)	52 (50 - 64)	0.7
Median BMI, (IQR)	29.1 (23.7 - 32.9)	26.4 (23.2 - 31.2)	0.4
Race			
Asian	2 (28.6%)	2 (33.3%)	0.8
Caucasian	5 (71.4%)	4 (66.7%)	
Ethnicity			
Hispanic or Latino	2 (28.6%)	0 (0.0%)	0.2
Non-Hispanic or Latino	5 (71.4%)	6 (100.0%)	
Median no. chemo regimens, (IQR)	1 (1 - 2)	1 (1 - 1)	0.3
0	*0 (0.0%)*	*1 (16.7%)*	
1	*4 (57.1%)*	*4 (66.7%)*	
2	*2 (28.6%)*	*0 (0.0%)*	
3-4	*1 (14.3%)*	*1 (16.7%)*	
Disease status at time of HIPEC			
Primary	1 (14.3%)	3 (50.0%)	0.3
Recurrent	6 (85.7%)	3 (50.0%)	
Median months since last regimen, (IQR)	8.5 (1.9 - 19.4)	4.0 (1.1 - 14.1)	0.5
Median PCI, (IQR)	6 (4 – 11)	7.5 (3 – 12)	1.0
Median OR time (hours), (IQR)	7.5 (6.4 – 9.0)	6.7 (6.7 – 8.2)	0.9
Median baseline creatinine (mg/dL), (IQR)	0.7 (0.5 - 0.8)	0.7 (0.7 - 0.7)	0.8
Median post-op creatinine (mg/dL), (IQR)	0.7 (0.5 - 0.8)	2.8 (2.0 - 4.0)	0.002

Supplementary Data 5: (A) GO BIO signaling pathways and (B) up- and down-regulated genes in patients with nephrotoxicity

A.

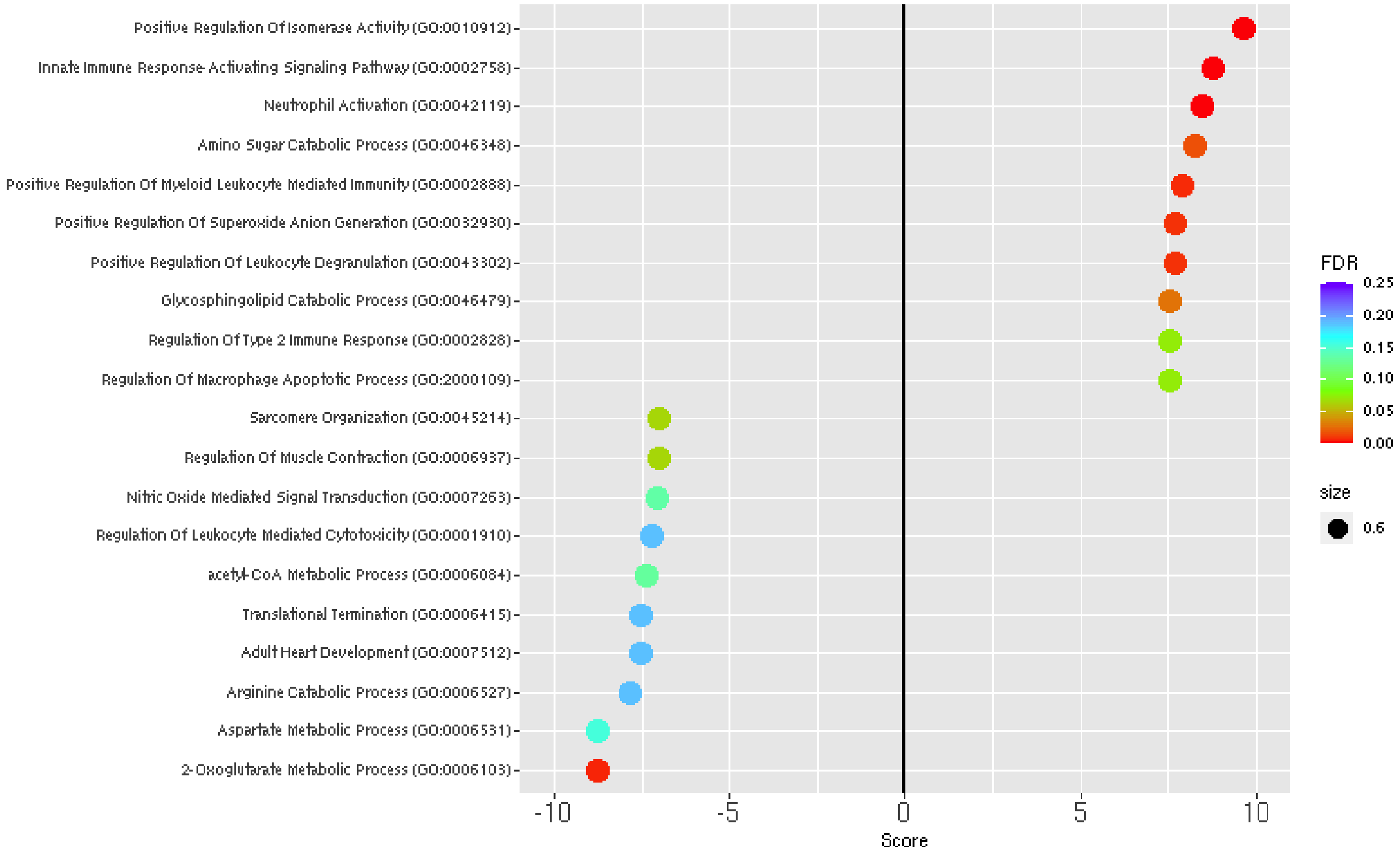

B.

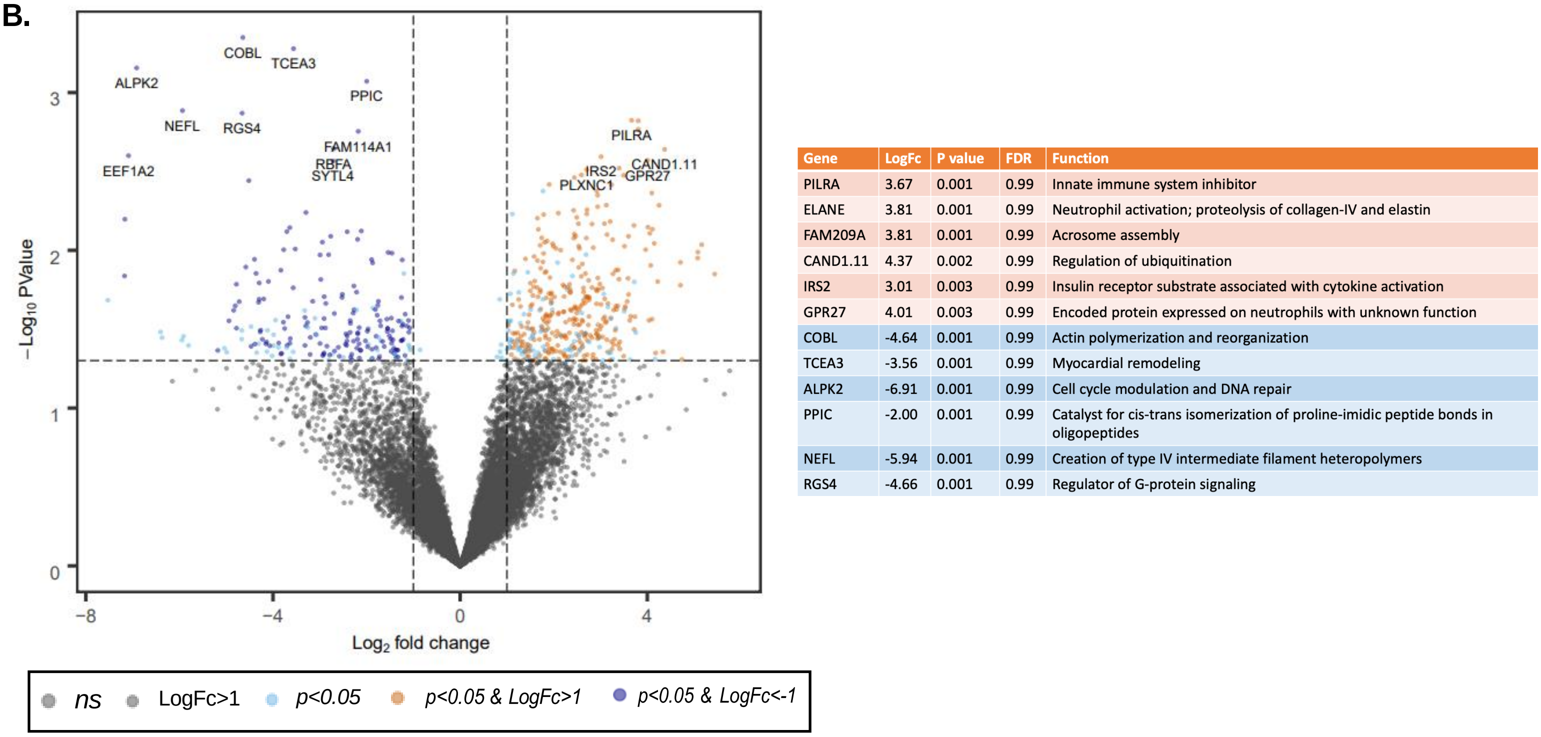

Gene	LogFc	P value	FDR	Function
PILRA	3.67	0.001	0.99	Innate immune system inhibitor
ELANE	3.81	0.001	0.99	Neutrophil activation; proteolysis of collagen-IV and elastin
FAM209A	3.81	0.001	0.99	Acrosome assembly
CAND1.11	4.37	0.002	0.99	Regulation of ubiquitination
IRS2	3.01	0.003	0.99	Insulin receptor substrate associated with cytokine activation
GPR27	4.01	0.003	0.99	Encoded protein expressed on neutrophils with unknown function
COBL	-4.64	0.001	0.99	Actin polymerization and reorganization
TCEA3	-3.56	0.001	0.99	Myocardial remodeling
ALPK2	-6.91	0.001	0.99	Cell cycle modulation and DNA repair
PPIC	-2.00	0.001	0.99	Catalyst for cis-trans isomerization of proline-imidic peptide bonds in oligopeptides
NEFL	-5.94	0.001	0.99	Creation of type IV intermediate filament heteropolymers
RGS4	-4.66	0.001	0.99	Regulator of G-protein signaling
